# Hepatitis B Immunization Among Healthcare Students: A Narrative Review of Occupational Risk, Seroprotection, and Immunization Management

**DOI:** 10.3390/vaccines14090732

**Published:** 2026-08-25

**Authors:** Lorenzo Ippoliti, Viola Giovinazzo, Luca Coppeta, Giuseppe Bizzarro, Cristiana Ferrari, Andrea Mazza, Agostino Paolino, Silvio Pallone, Matteo Pasanisi, Claudia Salvi, Greta Verno, Andrea Vischetti, Andrea Magrini

**Affiliations:** 1 Department of Biomedicine and Prevention, University of Rome Tor Vergata, 00133 Rome, Italy; 2 PhD Program in Social, Occupational and Medico-Legal Sciences, Department of Occupational Medicine, University of Rome Tor Vergata, 00133 Rome, Italy; 3 Faculty of Medicine, Saint Camillus International University of Health Sciences, 00131 Rome, Italy

**Keywords:** hepatitis B, HBV vaccination, healthcare students, occupational risk, seroprotection, non-responder, post-exposure prophylaxis

## Abstract

Background/Objectives: Healthcare students face an occupational risk of hepatitis B virus (HBV) exposure during clinical training, often many years after receiving their primary vaccination series in infancy or childhood. Progressive waning of anti-hepatitis B surface (anti-HBs) antibody titres, heterogeneous vaccination histories in international student cohorts, high rates of underreported needlestick injuries, and variable occupational health protocols across institutions raise important questions about the adequacy of pre-clinical immunization surveillance in this population. This review aimed to synthesize current evidence on hepatitis B immunization among healthcare students, focusing on occupational exposure risk, seroprotection and immunological memory, clinical management of low or absent anti-HB titres including post-exposure prophylaxis, and the specific challenges posed by multicultural academic settings. Methods: A narrative review was conducted through a systematic literature search of PubMed/MEDLINE and Scopus, covering publications from January 2000 to December 2025, supplemented by key seminal references. After the removal of duplicates and stepwise screening, 49 references were selected for final inclusion. Results: Needlestick and sharps injuries occur at measurable rates during clinical training, with medical students accounting for approximately 30% of occupational exposure events in some series, and with underreporting rates estimated at 19–80% across studies. Pooled seroprotection prevalence at clinical training entry is approximately 73.8% (95% CI 69.1–78.0%), with substantially lower rates among students vaccinated in infancy—as low as 28% at 16–20 years post-vaccination in longitudinal data—compared with adolescence immunization. The majority of students with non-protective titres retain immunological memory, with 90.9% (95% CI 87.7–93.3%) demonstrating an anamnestic response after a single booster dose. Post-exposure prophylaxis pathways depend critically on pre-existing immunological status, reinforcing the operational value of pre-clinical serological screening. International student cohorts present additional complexity due to heterogeneous vaccination schedules, documentation gaps, and variable natural immunity profiles. Conclusions: Systematic pre-clinical serological screening (anti-HBs, HBsAg, and anti-HBc), combined with evidence-based stepwise immunization management and structured educational interventions, is essential to protect healthcare students from occupational HBV exposure. The primary operational goal of screening is the identification of the approximately 5% of true non-responders who require tailored occupational health management. These findings support a proactive, integrated approach to hepatitis B immunization surveillance in healthcare education, aligned with the WHO 2030 viral hepatitis-elimination targets.

## 1. Introduction

Infection with the hepatitis B virus (HBV) remains one of the most significant preventable infectious diseases worldwide [1]. It is estimated that 254 million people were living with chronic HBV infection in 2022. Together, viral hepatitis B and C caused an estimated 1.3 million deaths that year, with HBV accounting for around 83% of this total [2,3]. HBV is a small, partially double-stranded DNA virus for which humans are the only relevant reservoir. This feature makes the infection potentially eradicable through sustained vaccination [4]. Based on this premise, the World Health Organization (WHO) has set targets to reduce new chronic HBV infections by 90% and HBV-related mortality by 65% by 2030, compared to 2015 levels [5]. A 2026 analysis estimated 283.64 million prevalent cases in 2021 with 431,960 deaths, and identified Eastern Europe, high-income North America and Central Asia as regions showing stable or increasing trends despite global progress [6].

Despite the availability of an effective vaccine since the early 1980s, HBV transmission continues to pose a public health challenge in populations at elevated risk of occupational exposure [7]. Healthcare workers are among the professions most frequently exposed to bloodborne pathogens through needlestick injuries, contact with contaminated sharps and exposure of mucous membranes to blood and body fluids. Accordingly, hepatitis B vaccination has been a cornerstone of the occupational health policy in healthcare settings for decades [8,9].

Within this broader picture, healthcare students represent a specific subpopulation of concern. During clinical training, students on medical, nursing, dental and allied health programmes begin to face occupational risks similar to those of qualified practitioners, despite having completed their primary HBV vaccination series in infancy or childhood many years earlier. Anti-hepatitis B surface antibody (anti-HBs) levels are known to decline over time, and vaccination-coverage statistics alone do not accurately reflect the proportion of students who retain detectable protective titres by the time they enter clinical placements [10,11]. The increasing internationalisation of higher education exacerbates this uncertainty, as universities are enrolling a more diverse range of students with different vaccination schedules, documentation standards and natural exposure histories [12].

A critical conceptual distinction must be established at the outset: a decline in circulating anti-HBs below the conventional protective threshold of 10 mIU/mL does not, in most cases, indicate loss of protection. The majority of previously vaccinated individuals retain immunological memory and mount a rapid anamnestic response upon re-exposure to HBV antigens [10,11]. The primary operational goal of pre-clinical serological screening is therefore the detection of the minority of true non-responders—approximately 5% of previously vaccinated individuals—who lack both circulating antibodies and immunological memory and who are therefore at genuine occupational risk [10,11].

This narrative review aimed to synthesise the available evidence on hepatitis B immunisation among healthcare students. The review addressed three key areas: (i) seroprotection and the persistence of long-term immunity in this population, (ii) the evidence-based management of students with low or absent anti-HBs titres, and (iii) the specific challenges posed by multicultural academic environments. Throughout, practical implications for occupational health services operating within university settings are discussed.

## 2. Materials and Methods

This article is a narrative review. A literature search was performed in PubMed/MEDLINE and Scopus, covering publications from January 2000 to December 2025. Where they provided foundational evidence, seminal studies predating this period were also considered. The reference lists of all included articles were screened for additional relevant sources.

The search strategy employed the following terms in various combinations: (“hepatitis B” OR “HBV”) AND (“vaccination” OR “immunization” OR “anti-HBs” OR “seroprotection” OR “long-term immunity” OR “waning immunity” OR “booster” OR “non-responder” OR “anamnestic response” OR “antibody persistence”) AND (“healthcare students” OR “medical students” OR “nursing students” OR “dental students” OR “health profession students” OR “healthcare workers” OR “occupational health”) AND (“needlestick” OR “sharps injury” OR “occupational exposure” OR “post-exposure prophylaxis” OR “bloodborne pathogens” OR “fitness for duty” OR “multicultural” OR “international students”). No language restriction was applied.

The initial database search yielded 312 records from PubMed/MEDLINE and 187 from Scopus (total *n* = 499). After removal of 87 duplicates, 412 records underwent title and abstract screening. Of these, 89 were retrieved for full-text evaluation. Following application of inclusion and exclusion criteria, 49 references were selected for final inclusion, of which 34 were identified as primary sources for the thematic synthesis in the Results section; the remaining 15 served as background references in the Introduction and Discussion.

Articles were considered eligible for inclusion if they addressed one or more of the following: seroprotection rates or antibody persistence following hepatitis B vaccination in healthcare students or workers; the clinical management of low or absent anti-HB titres; the risk of occupational exposure and post-exposure prophylaxis in healthcare trainees; or immunisation challenges in multicultural or international academic settings. Opinion pieces and case reports were excluded unless they provided background data of direct relevance; studies focusing exclusively on therapeutic HBV management were excluded.

## 3. Results

The following sections summarise the available evidence across five themes: occupational exposure risk; the trajectory of vaccine-induced immunity; seroprotection rates in healthcare students; clinical management of low or absent anti-HB titres, including post-exposure prophylaxis; and challenges in multicultural academic environments. A final section addresses the medicolegal implications of immunization status in the context of fitness for clinical duty.

### 3.1. Occupational Exposure Risk in Healthcare Students

Healthcare students face measurable occupational risk of exposure to bloodborne pathogens during clinical training [13,14,15]. Studies examining occupational exposure events in tertiary hospitals have consistently identified medical students as among the highest-risk groups, accounting for approximately 30% of all reported needlestick and sharps injuries (NSIs) in some series, alongside nurses and junior doctors [16]. Annual NSI prevalence has been reported as high as 13.9% in Australian nursing students [17] and approximately 18% in Italy [18], with considerable heterogeneity attributable to differences in surveillance systems, clinical activity volume and reporting culture [19]. NSIs predominantly occur during injection administration, venepuncture and surgical assistance, and are most commonly located on the fingers; hospital wards and operating theatres account for the majority of incidents [16].

A notable and persistent challenge is underreporting. Studies estimate that 19% to over 80% of NSIs sustained by trainees go unreported, with nursing students exhibiting particularly high non-disclosure rates [20,21]. Barriers include the perception that the injury was minor, unfamiliarity with reporting procedures, time pressure, and—specific to the student context—fear that reporting might negatively affect academic evaluations or relationships with supervisors [21]. Unreported exposures may result in delayed or absent post-exposure evaluation and prophylaxis, with potential consequences for both the student and the patient. Occupational health services should therefore address underreporting through clear, non-punitive reporting pathways; proactive communication of procedures at the start of each clinical rotation; and explicit institutional reassurance that reporting carries no academic consequences.

### 3.2. Vaccine-Induced Immunity: Mechanisms and Expected Trajectory

The standard primary HBV vaccination series consists of three doses at 0, 1 and 6 months. An anti-HB concentration of ≥10 mIU/mL, measured four to eight weeks after completing the series, is internationally accepted as the threshold for seroprotection [22]. Initial seroconversion rates exceed 95% in healthy individuals completing the full series, declining with increasing age at vaccination: approximately 92% in adults under 40 years and 84% in those over 40 years [10].

Long-term protection is primarily attributed to the persistence of immunological memory rather than to circulating antibody levels alone [23]. Follow-up studies spanning 30 and 35 years have shown that protection against clinical HBV infection is maintained in the majority of vaccinated individuals even after anti-HBs becomes undetectable, owing to memory B cells and long-lived plasma cells capable of mounting a rapid anamnestic response upon re-exposure [24,25]. Following primary infant vaccination, the rate of antibody decline has been estimated at approximately −42.39 mIU/mL per year in a Thai cohort, with seroprotection rates of 28%, 51.7% and 60% at 16–20, 21–25 and 26–28 years post-vaccination, respectively [26]. The apparently non-linear pattern likely reflects survivor bias and intermediate revaccination events rather than a true increase in circulating antibodies, and should be interpreted with caution.

### 3.3. Seroprotection and Waning Immunity in Healthcare Students

Despite the high vaccination coverage reported in countries with long-standing universal childhood immunisation programmes, the proportion of healthcare students with protective anti-HB levels at the time of pre-clinical screening varies considerably across studies. A systematic review and meta-analysis of 46 studies involving 52,749 healthcare students from highly developed countries found a pooled seroprotection prevalence of 73.8% (95% confidence interval [CI]: 69.1–78.0) at the pre-exposure assessment. This indicates that over one in four students had anti-HB levels below the protective threshold when they started clinical training [11]. Meta-regression analyses from the same study identified time elapsed since vaccination and the proportion of students vaccinated in infancy rather than adolescence as significant negative predictors of seroprotection. The main characteristics and seroprotection rates of key individual studies are summarised in Table 1.

Findings from individual cohort studies are broadly consistent with this pooled estimate, while illustrating substantial heterogeneity in terms of setting, geographic context and time since vaccination. A four-year retrospective study of 2028 healthcare trainees at a central Italian hospital reported an overall seroprotection prevalence of 50.7%. Protective immunity was observed in 79.2% of students who were vaccinated during adolescence, compared to 44.6% of those who were vaccinated in infancy (*p* < 0.001). This result reinforces the idea that age at vaccination is a key determinant of residual antibody levels [29]. A separate cross-sectional study of 507 health profession students at an international, multi-ethnic university found overall seroprotection in only 55.0% of participants, with lower rates among those vaccinated in infancy and born outside Europe after adjusting for time since the last dose [12]. In a South African university setting, baseline screening before a booster intervention found that 56% of healthcare students were non-immune (defined as anti-HBs < 10 mIU/mL per WHO [22]), a figure consistent with lower rates reported in European cohorts. This underscores the fact that inadequate seroprotection at the start of clinical training is not confined to high-income Western settings [30]. Taken together, these studies suggest that the proportion of students who can be assumed to be seroprotected without testing is determined by vaccination timing, geographic and demographic background, and time since the primary series.

Antibody waning is a well-documented and expected phenomenon, rather than being evidence of vaccine failure. A study of 734 Italian healthcare workers and medical students who were vaccinated in infancy or adolescence found that 12.0% of subjects (88/734) had a non-protective anti-HB titer. Of those who received a booster dose, almost 90% had a protective titer one month later, indicating that the initial low titers were due to the physiological decline in antibodies over time rather than vaccine failure [10]. A national meta-analysis of 19 studies on Italian healthcare workers estimated an overall hepatitis B sero-susceptibility prevalence of 27.1% (95% CI: 23.2–31.7), with a markedly lower risk among those vaccinated in adolescence compared to those vaccinated in infancy (relative risk: 0.30; 95% CI: 0.25–0.37) [27]. A separate cohort study of 539 healthcare workers who had been vaccinated up to 30 years previously found that the rate of subsequent anti-HB loss differed sharply according to the magnitude of the initial post-vaccination response: there were 52.1 losses per 1000 person-years among poor responders (initial anti-HBs 10–99 mIU/mL), compared with 11.3 among moderate responders, and just 1.4 among good responders (≥1000 mIU/mL). This identified the peak post-vaccination titer as the strongest predictor of long-term seroprotection [28]. These findings are consistent with longitudinal quantitative data from Thailand, where the overall rate of anti-HB decline following primary infant vaccination was estimated at −42.39 mIU/mL per year, and with the immunological memory framework. The meta-analysis by Rahmani et al. found that 90.9% (95% CI 87.7–93.3) of students with non-protective anti-HBs at pre-exposure screening mounted a demonstrable anamnestic response after a single booster dose. Only 5.0% (95% CI 2.1–11.5) were classified as true non-responders after a complete second vaccination cycle [11,26]. Accordingly, current WHO and ACIP guidance holds that a decline in anti-HBs below 10 mIU/mL, in the absence of documented exposure, does not indicate susceptibility to infection [22,24].

### 3.4. Management of Low or Absent Anti-HB Titres

The most commonly encountered finding in healthcare students during pre-clinical occupational health screening is the detection of anti-HBs below 10 mIU/mL. This calls for a stepwise, evidence-based response rather than assuming susceptibility. An algorithm synthesised from current guidance is summarised in Table 2.

For students with a documented complete primary series and non-protective anti-HBs levels, guidelines recommend a single booster dose followed by serological retesting after four to eight weeks [31,33]. A retrospective observational study in Japan validated this stepwise approach, demonstrating that vaccination-history-guided revaccination yielded effective seroconversion in the majority of participants and was superior to uniform revaccination strategies [34]. Students failing to respond to the booster are classified as potential primary non-responders and should receive a second full three-dose series; those remaining seronegative thereafter are classified as definitive non-responders [11,27,28]. These individuals require post-exposure prophylaxis counselling, access to hepatitis B immunoglobulin (HBIG), and may be subject to institutional risk-based restrictions on clinical activities.

For the approximately 5% of definitive non-responders, multivalent vaccine formulations incorporating preS1 and preS2 surface antigens produced in mammalian cell lines have demonstrated efficacy where conventional yeast-derived vaccines have failed [35]. These formulations are licensed in several countries and represent a clinically relevant option for this subgroup.

Occupational exposure to blood or body fluids requires prompt evaluation; HBV-specific post-exposure prophylaxis (PEP) should be administered where indicated. The student’s immunological status at the time of the incident is the primary determinant of the appropriate management pathway. Students with documented seroprotection (anti-HBs ≥ 10 mIU/mL) do not require HBV-directed PEP regardless of source HBsAg status [32]. For students with unknown or non-protective anti-HBs, or confirmed non-responders, HBIG should be administered as soon as possible if the source is HBsAg-positive or unknown, ideally within 12–24 h and no later than seven days [36,37,38,39]. Definitive non-responders should receive two doses of HBIG (0.06 mL/kg) when the source is HBsAg-positive [36,37,38,39]. Recommended PEP pathways by immunological scenario are summarised in Table 3.

### 3.5. Challenges in Multicultural and International Student Populations

Internationalisation of higher education introduces substantial heterogeneity into the immunization profiles of healthcare student cohorts. Students arrive from countries with different HBV epidemiology, vaccination schedules (birth-dose, infant, or adolescent programmes), vaccine quality, cold chain standards, and documentation practices, complicating reliance on self-reported vaccination history [40,41]. HBV vaccination coverage among healthcare workers has been estimated at 18% in parts of Africa, 77% in Australia and New Zealand, 82% in Egypt, and 80% in South Africa, illustrating the scale of disparity that international student cohorts may introduce [42,43,44]. Seroprevalence of natural HBV immunity in students from intermediate- or high-endemicity regions further complicates the interpretation of anti-HBs results, since a positive titre may reflect vaccination or past infection.

Data from a multi-ethnic Italian university cohort confirm this directly: students of non-European origin showed significantly lower seroprotection than European-born counterparts even after adjusting for age at vaccination and time since last dose [12]. Similar findings from South Africa—56% non-immunity despite national infant vaccination programmes—demonstrate that the gap between vaccination coverage and actual seroprotection at training entry is a global phenomenon [30]. Anti-HBc positivity in the absence of HBsAg indicates past resolved HBV infection. Individuals with evidence of prior infection may retain partial or complete immunity—including T-cell-mediated responses—and should be evaluated following the same serological screening algorithm applied to all other students: if anti-HB ≥ 10 mIU/mL is documented, seroprotection is confirmed; if anti-HB is below this threshold, clinical judgement guided by institutional occupational health protocols should determine whether further intervention is warranted.

Universal three-marker serological screening (anti-HBs, HBsAg, anti-HBc) at clinical training entry, irrespective of self-reported history, classifies students into four operationally distinct groups: vaccine-induced seroprotection (no action required); susceptible (vaccination needed); naturally immune (document and monitor); and chronically infected (refer to hepatology) [29]. For students without vaccination documentation, administration of the complete primary series with subsequent serological verification is the recommended pragmatic approach.

### 3.6. Fitness for Duty Assessment: The Italian Framework and Its International Relevance

In Italy, the occupational health assessment of healthcare students and workers is governed by Legislative Decree 81/2008 (the Consolidated Act on Occupational Health and Safety). Article 279 of this decree stipulates that workers exposed to biological agents must undergo health surveillance, and that effective vaccines must be made available to those who are not already immune. The occupational physician (medico competente) is responsible for determining fitness for duty (giudizio di idoneità alla mansione specifica) in relation to the biological risk profile of the clinical role. In the context of hepatitis B, vaccination status and serological response directly inform this assessment. A healthcare student who is a documented non-responder or who refuses vaccination without a valid medical contraindication may receive a restricted fitness judgement. For example, they may be excluded from invasive procedures carrying a high risk of blood exposure. Alternatively, if no alternative clinical assignment is practicable, they may be given a temporary judgement of unfitness for the specific role [28,32]. This medico-legal mechanism, which links immunisation status directly to access to clinical training, has no direct equivalent in most other European countries, where institutional policies on vaccination for healthcare students are highly variable and often non-binding. Nevertheless, the underlying principle—that documented susceptibility to a preventable blood-borne pathogen should have operational consequences within an occupational health framework—is an increasingly discussed topic in the international literature, particularly in the context of strengthening occupational vaccination programmes for healthcare trainees [43]. The Italian model may therefore serve as a useful reference for institutions and regulators in other countries seeking to formalise the relationship between pre-clinical immunisation screening and access to clinical placements.

## 4. Discussion

Universal childhood vaccination, while highly effective at the population level, does not guarantee uniform seroprotection among healthcare students at the time of clinical training entry. Pooled data indicate that approximately one in four students in highly developed countries presents with anti-HBs below the protective threshold; individual cohort studies report rates of 50–56% in settings where a large proportion of students were vaccinated in infancy or trained outside their country of origin [10,11,12,29]. This contrasts sharply with the reassurance suggested by high national vaccination-coverage figures and has direct implications for how occupational health services design pre-clinical screening programmes.

The high prevalence of anamnestic responses—exceeding 90% after a single booster—indicates that low anti-HBs at screening primarily reflect waning circulating antibodies rather than lost immunity [11]. This distinction is clinically fundamental: the stepwise approach outlined in Table 2 is justified precisely because only approximately 5% of previously vaccinated individuals—those who remain seronegative after a booster dose and a complete second three-dose vaccination series—represent true definitive non-responders who are genuinely susceptible to HBV acquisition upon occupational exposure. Blanket revaccination of all students with low titres is unnecessary, costly, and not supported by the available evidence.

The findings on multicultural cohorts raise a question the literature has not yet fully answered: does lower seroprotection among internationally born students reflect documentation and timing gaps addressable with better records, or more durable differences in vaccine type, schedule adherence, or population-level immune response? Existing studies are largely cross-sectional and drawn from single institutions, limiting causal inference [12,29].

Underreporting of NSIs is a significant and persistent problem with direct implications for post-exposure management. The range of 19–80% across studies reflects both true variability and methodological differences in reporting assessment; the consistent finding that fear of academic consequences is a specific barrier in student populations [45] points to an institutional culture issue requiring proactive intervention rather than passive information provision. In particular, unfamiliarity with reporting procedures and time pressure were identified as key barriers at the point of injury. This suggests that institutional interventions should focus on cultural change and streamlining the reporting process itself. For instance, this could be achieved by providing digital reporting tools that are accessible during clinical shifts. Additionally, brief mandatory training on post-exposure pathways could be integrated into the induction programme of each clinical rotation.

Beyond the physical risk and the operational consequences of underreporting, needlestick injuries carry a significant psychological burden that is rarely addressed in occupational health programmes for healthcare students. The psychological consequences of NSIs represent an underaddressed dimension of occupational risk. Over 80% of healthcare workers sustaining an occupational exposure report immediate anxiety; post-traumatic stress disorder following high-risk exposures has been documented [46]. Pre-existing documented seroprotection substantially reduces this burden by enabling immediate reassurance that no HBV-directed prophylaxis is required.

Educational interventions targeting safe sharps handling, reporting culture and post-exposure procedures have demonstrated measurable effectiveness [47]. A Cochrane-registered systematic review found structured training significantly improved post-exposure knowledge and, in one controlled study, raised hepatitis B vaccination uptake from 71.4% to 91.8% [47]. Knowledge alone is insufficient; programmes must incorporate supervised practical components and explicit instruction on reporting culture [48].

From a health economics perspective, modelled incremental cost-effectiveness ratios of $144,000 to over $800,000 per QALY under assumptions of 95% long-term protection approach acceptable thresholds when real-world seroprotection rates closer to 68% are applied [49]—a scenario consistent with the data reviewed here. Reframing the question from ‘Is screening cost-effective compared to doing nothing?’ to ‘What is the cost of not knowing a student’s serological status at the time of an occupational incident?’ may be the more policy-relevant approach.

### Limitations

Several limitations of the available evidence base should be acknowledged when interpreting the findings of this review. First, the majority of primary studies are cross-sectional and conducted at single institutions, which limits causal inference and generalisability. The substantial heterogeneity in reported seroprotection rates across studies—ranging from approximately 44% to 89% in the cohorts reviewed—likely reflects genuine differences in population characteristics and vaccination timing, but also methodological variability that cannot be disentangled from cross-sectional data alone. Second, serological assays for anti-HBs are not standardised across studies. Differences in assay platform, calibration standards, and operational cut-off definitions introduce systematic measurement heterogeneity that limits direct comparability of seroprotection rates across cohorts, even when studies nominally apply the same 10 mIU/mL threshold. Third, survivor bias represents a specific concern in longitudinal data, most notably in the Thai cohort [26], where the counterintuitive increase in seroprotection rates across later time intervals most likely reflects progressive loss to follow-up of lower-titre individuals and unaccounted intermediate revaccination events, rather than true antibody recovery. Fourth, self-reported vaccination history—the primary basis for infant versus adolescent vaccination stratification in several key studies—is subject to recall and documentation bias, and may systematically misclassify individuals in ways that distort group-level seroprotection estimates. Finally, the evidence base is geographically concentrated in high-income European settings, with limited representation from other regions, which restricts the generalisability of the pooled estimates to more diverse healthcare education contexts.

Future priorities include prospective multinational longitudinal studies tracking anti-HB kinetics in cohorts vaccinated under contemporary schedules, and comparative evaluations of screening strategies across institutions with differing student demographics. Universal three-marker serological screening at clinical training entry remains the most defensible basis for individualized immunization decisions.

## 5. Conclusions

Hepatitis B immunization among healthcare students is a multidimensional occupational health issue that extends beyond vaccine coverage statistics. Despite high childhood-vaccination coverage, approximately one in four students—and up to one in two among those vaccinated in infancy—present with non-protective anti-HB titres at clinical training entry. The majority retains immunological memory and responds to a single booster dose; evidence-based stepwise management identifies approximately 5% of true non-responders who require specific counselling and tailored post-exposure prophylaxis planning.

Needlestick injuries occur at measurable rates and are substantially underreported, compromising timely post-exposure management. Structured educational interventions and non-punitive reporting pathways are effective and should be systematically integrated into pre-clinical training. International student cohorts require flexible screening protocols that account for heterogeneous vaccination histories and natural immunity profiles.

Universal three-marker serological screening (anti-HBs, HBsAg, anti-HBc) at clinical training entry, combined with evidence-based immunization management, provides the most reliable basis for protecting healthcare students, determining post-exposure prophylaxis pathways, and informing fitness for clinical duty assessments. These findings support a proactive, integrated approach to hepatitis B immunization surveillance aligned with the WHO 2030 viral hepatitis elimination targets.

## Figures and Tables

**Table 1:** Summary of key studies on seroprotection rates among healthcare students and workers.

Author (s), Year	Country	Study Design	n	Population	Overall Seroprotection (Anti-HBs ≥ 10 mIU/mL)	Vaccinated in Infancy	Vaccinated in Adolescence	Key Predictor (s)
Rahmani et al., 2022 [11]	Multi-country	SR & meta-analysis (46 studies)	52,749	Healthcare students	73.8% (95% CI 69.1–78.0)	Lower (meta-regression)	Higher (meta-regression)	Time since vaccination; age at vaccination
Bianchi et al., 2023 [27]	Italy	SR & meta-analysis (19 studies)	NR	Healthcare workers	Sero-susceptibility 27.1% (95% CI 23.2–31.7)	Higher sero-susceptibility (RR vs. adolescence: 0.30, 95% CI 0.25–0.37)	Lower sero-susceptibility	Age at primary vaccination
Coppeta et al., 2019 [10]	Italy	Cross-sectional	734	HCW + medical students	88.0% (non-protective: 12.0%, 88/734)	Not stratified	Not stratified	~20 years post-vaccination; ~90% booster response rate
Cocchio et al., 2021 [28]	Italy	Cohort (≤30 years follow-up)	539	Healthcare workers	Progressive decline by responder category	Not stratified	Not stratified	Peak post-vaccination titre: loss rates 52.1/1000 PY (poor) vs. 1.4/1000 PY (good responders)
Di Giampaolo et al., 2025 [29]	Italy	Retrospective cohort (4 years)	2028	Healthcare trainees	50.7%	44.6%	79.2% (*p* < 0.001)	Age at vaccination; time since vaccination
Ippoliti et al., 2025 [12]	Italy (international university)	Cross-sectional	507	Multi-ethnic health profession students	55.0%	Lower (significant)	Higher (significant)	Age at vaccination; geographic origin
Makan et al., 2023 [30]	South Africa	Prospective interventional	180	Healthcare students	44% seroprotected at baseline (56% non-immune)	Not stratified	Not stratified	Non-immunity prevalent despite national infant vaccination programme; high booster response
Phattraprayoon et al., 2022 [26]	Thailand	Retrospective cohort	464	Medical students and HCW	Variable by time interval: 28% (16–20 years), 51.7% (21–25 years), 60% (26–28 years)	Primary vaccination in infancy	N/A	Antibody decline rate: −42.39 mIU/mL/year; survivor bias and intermediate revaccination likely affect interval rates

Abbreviations: HCW, healthcare workers; NR, not reported; PY, person-years; RR, relative risk; CI, confidence interval; SR, systematic review. Anti-HBs ≥ 10 mIU/mL is the internationally accepted threshold for seroprotection. Studies listed in chronological order of publication.

**Table 2:** Suggested stepwise management of hepatitis B vaccination status in healthcare students, synthesised from the WHO [22], CDC/ACIP [31], and Italian Legislative Decree 81/2008, Article 279 [32]).

Step	Action/Decision	Note
Step 1	Obtain vaccination history and measure anti-HBs, HBsAg, and anti-HBc in all incoming healthcare students	Perform at clinical training entry, regardless of self-reported vaccination history. Include anti-HBc to identify natural immunity or chronic infection
Step 2a	Anti-HBs ≥ 10 mIU/mL: document seroprotection; no further intervention required	No further vaccination required. Document result in occupational health record
Step 2b	Anti-HBs < 10 mIU/mL, with documented complete primary series: administer a single booster dose; retest anti-HBs after 4–8 weeks	Applicable to students with documented complete primary series only. If vaccination history is unknown or incomplete, proceed directly to full primary series
Step 3a	Post-booster anti-HBs ≥ 10 mIU/mL: confirm immunological memory; document seroprotection	Confirms persistence of immunological memory. Seroprotection expected to be durable; no routine booster required thereafter
Step 3b	Post-booster anti-HBs < 10 mIU/mL: complete a second full three-dose vaccination series	Administer standard 3-dose series (0, 1, 6 months). Retest anti-HBs 4–8 weeks after final dose
Step 4a	After second series, anti-HBs ≥ 10 mIU/mL: document seroprotection	Document seroprotection. Consider periodic monitoring in high-exposure settings
Step 4b	After second series, anti-HBs < 10 mIU/mL: classify as a non-responder; consider a preS1/preS2-containing vaccine; provide specific post-exposure prophylaxis counselling	PreS1/PreS2-containing vaccines (third generation) may achieve seroprotection in a proportion of non-responders. Restrict high-risk clinical activities according to institutional policy
Special case	Anti-HBc positive, HBsAg negative: natural immunity is documented. Anti-HBc positive, HBsAg positive: refer to hepatology for chronic HBV management	Anti-HBc+/HBsAg– students do not require vaccination. Anti-HBc+/HBsAg+ students should be referred to hepatology before commencing clinical activities

**Table 3:** Post-exposure management of HBV occupational exposure based on the student’s immune status and source HBsAg status (based on U.S. PHS guidelines [37], CDC/ACIP [31], and Senoo-Dogbey et al. [36]).

Exposed Student’s HBV Immune Status	Source HBsAg Positive	Source HBsAg Negative	Source HBsAg Unknown
Documented seroprotection (anti-HBs ≥ 10 mIU/mL)	No PEP required	No PEP required	No PEP required
Vaccinated; anti-HBs unknown or not previously tested	Test anti-HBs; if <10 mIU/mL, administer HBIG and revaccinate	Test anti-HBs; manage according to result	Test anti-HBs; manage according to result
Vaccinated, documented non-responder (anti-HBs < 10 mIU/mL after two complete vaccine series)	HBIG × 2 (1 month apart)	No PEP required	Assess exposure risk; manage according to institutional protocol
Unvaccinated or incompletely vaccinated	HBIG + initiate HBV vaccination	Initiate HBV vaccination	Initiate HBV vaccination
Natural immunity (anti-HBc positive, HBsAg negative)	No PEP required	No PEP required	No PEP required
Chronic HBV infection (HBsAg positive)	No PEP required *	No PEP required *	No PEP required *

Abbreviations: HBsAg, hepatitis B surface antigen; anti-HBs, antibody to hepatitis B surface antigen; anti-HBc, antibody to hepatitis B core antigen; HBIG, hepatitis B immune globulin; PEP, post-exposure prophylaxis. * Individuals with chronic HBV infection do not require post-exposure prophylaxis and should receive appropriate clinical follow-up.

## Data Availability

No new data were generated or analysed in support of this research. This article is a narrative review based exclusively on previously published studies, all of which are cited in the reference list.

## Abbreviations

The following abbreviations are used in this manuscript:

ACIP	Advisory Committee on Immunization Practices
anti-HBc	Antibody to hepatitis B core antigen
anti-HBs	Hepatitis B surface antibodies
CDC	Centers for Disease Control and Prevention (United States)
CI	Confidence interval
D.Lgs.	Decreto Legislativo (Italian Legislative Decree)
HBIG	Hepatitis B immunoglobulin
HBsAg	Hepatitis B surface antigen
HBV	Hepatitis B virus
HCW	Healthcare worker
mIU/mL	Milli-international units per millilitre
NR	Not reported
NSI	Needlestick and sharps injury
PEP	Post-exposure prophylaxis
PY	Person-years
QALY	Quality-adjusted life year
RR	Relative risk
WHO	World Health Organization
